# Epidemiology and trends for Caesarean section births in New South Wales, Australia: A population-based study

**DOI:** 10.1186/1471-2393-11-8

**Published:** 2011-01-20

**Authors:** Efty P Stavrou, Jane B Ford, Antonia W Shand, Jonathan M Morris, Christine L Roberts

**Affiliations:** 1 Clinical and Population Perinatal Research, Kolling Institute of Medical Research, University of Sydney at Royal North Shore Hospital, St Leonards, NSW, 2065, Australia; 2 Department of Obstetrics and Gynaecology, University of Sydney at Royal North Shore Hospital, St Leonards, NSW, 2065, Australia

## Abstract

**Background:**

Caesarean section (CS) rates around the world have been increasing and in Australia have reached 30% of all births. Robson's Ten-Group Classification System (10-group classification) provides a clinically relevant classification of CS rates that provides a useful basis for international comparisons and trend analyses. This study aimed to investigate trends in CS rates in New South Wales (NSW), including trends in the components of the 10-group classification.

**Methods:**

We undertook a cross-sectional study using data from the Midwives Data Collection, a state-wide surveillance system that monitors patterns of pregnancy care, services and pregnancy outcomes in New South Wales, Australia. The study population included all women giving birth between 1^st ^January 1998 and 31st December 2008. Descriptive statistics are presented including age-standardised CS rates, annual percentage change as well as regression analyses.

**Results:**

From 1998 to 2008 the CS rate in NSW increased from 19.1 to 29.5 per 100 births. There was a significant average annual increase in primary 4.3% (95%CI 3.0-5.7%) and repeat 4.8% (95% CI 3.9-5.7%) CS rates from 1998 to 2008. After adjusting for maternal and pregnancy factors, the increase in CS delivery over time was maintained. When examining CS rates classified according to the 10-group classification, the greatest contributors to the overall CS rate and the largest annual increases occurred among nulliparae at term having elective CS and multipara having elective repeat CS.

**Conclusions:**

Given that the increased CS rate cannot be explained by known and collected maternal or pregnancy characteristics, the increase may be related to differences in clinical decision making or maternal request. Future efforts to reduce the overall CS rate should be focussed on reducing the primary CS rate.

## Background

Caesarean section (CS) rates around the world have been increasing, with current rates in Australia comparable to other industrialised nations such as United States and Canada [1-8]. The rise in the CS rate in the US has been most rapid since 2000 and has been attributed to an increase in the primary CS rate and a decrease in the vaginal birth after caesarean rate [4,6]. International concern over such increases have prompted the World Health Organisation to suggest that CS rates should not exceed 15% [9], with some evidence indicating caesarean section rates above 15% are not associated with additional reduction in maternal and neonatal mortality and morbidity [10]. The decision to perform a primary CS has important implications for maternal morbidity in the current pregnancy and mode of delivery and maternal morbidity in subsequent pregnancies [11-13].

Understanding population trends in CS rates, including trends in primary versus repeat CS, and potential drivers of these trends provide important insights into target areas for reducing the overall CS rate. Previous research has highlighted potential reasons for the increasing CS rate including maternal request[14,15] and associated ethical and litigious issues,[15-17] obesity[18,19] and increasing maternal age [20-22].

Population health data have been used to contrast CS rates in women with 'indicated medical risk' compared to women with 'no indicated risk'. Such research has demonstrated that primary CS rates appear to be unrelated to the medical risk profile of mothers, and change in a similar pattern to the overall CS rates [23]. The CS rate for mothers with 'no indicated risk', that is, for term, cephalic presenting, singleton births with no reported medical risk factors in the mother, has also increased over time. This research has been hampered by reliance on recording of factors indicating medical risk, many of which may be underestimated using reported data [6,24,25].

To allow for analysis of more clinically relevant characteristics, Robson[26] proposed a new classification system, the Robson Ten Group Classification System (10-group classification). This classification system is a mutually exclusive, totally exhaustive prospectively determined classification system [17,26]. The characteristics of a woman's pregnancy (which are reliably reported in population data), rather than the reason for a CS, form the basis for the classifications. The characteristics of the pregnancy used for the 10-group classification are (i) single or multiple pregnancy; (ii) nulliparous, multiparous or multiparous with a previous CS; (iii) cephalic, breech presentation or other lies; (iv) spontaneous or induced labour and (v) term or preterm births. It has been used in single-institution studies, jurisdictional and national registries and recently with international comparisons [2,5,8,27-29]. The increase in reporting using this classification system across a range of population data should facilitate understanding of the groups of women around the world in whom the CS rate is increasing.

Australian maternity care includes both public and private care; all women are covered by national health insurance, which provides free maternity care for patients in public hospitals, however about one third of women use private medical insurance or pay for private obstetric care. Public care is provided in a range of settings from small rural hospitals where care may be provided by GP obstetricians, to district hospitals with limited neonatal care facilities to tertiary obstetric hospitals with neonatal intensive care units. Midwifery care within the public hospital system may be provided by rostered hospital midwives or less commonly it involves continuity of care with antenatal, intrapartum and postpartum care by a named midwife (caseload), group of midwives (team midwifery), with support from the hospital obstetric staff when required.

The aim of this study was to investigate trends in CS rates in New South Wales (NSW), classified as primary or repeat caesarean section and classified according to the 10-group classification. Trends in CS rates were adjusted for known maternal and obstetric factors which may have changed over the study period and affected intervention rates.

## Methods

The Midwives Data Collection (referred to as 'birth data') is a state-wide surveillance system that monitors patterns of pregnancy care, services and pregnancy outcomes. It covers all births in New South Wales, that is, all livebirths and stillbirths of at least 20 weeks gestation or 400 g birth weight. The study population consisted of all women who gave birth in NSW from 1^st ^January 1998 to 31^st ^December 2008 inclusive. This study was approved by the NSW Population and Health Services Research Ethics Committee.

Variables of interest from the birth data included maternal age, year of delivery, parity, total number of previous caesareans, delivery mode, onset of labour, gestational age, birth presentation, hospital level, maternal smoking during pregnancy, birthweight (from which weight-for-gestational age [percentiles] were derived), any maternal hypertension (gestational, pre-eclampsia or chronic) and any maternal diabetes (gestational or pre-gestational). Term births were defined as births occurring at or after 37 weeks gestation. Hospitals were grouped according to the level of obstetric care provided: small rural (care provided by general practitioners and midwives), district (care available from rostered specialist obstetricians), tertiary obstetric (tertiary obstetric care with or without tertiary neonatal care), and private hospitals [30].

Primary CS was classified as the first caesarean procedure for the mother, regardless of parity. Repeat CS were identified where the number of previous caesareans was at least one; this has been found to be reliably reported on the birth data [31]. In order to examine more clinically relevant groupings for CS rates, analyses based on the 10-group classification were undertaken. For the purposes of this paper, an arbitrarily-defined 'short-form description' was adopted when referring to each group (Table 1).

**Table 1:** Ten-group classification

10-Group Classification:	Short-form description:
Nulliparous, single vertex, ≥37 wk, spontaneous labour -1	Nullip Term Spontaneous
Nulliparous, single vertex, ≥37 wk, Induced or CS wi No Labour-2	Nullip Term Elective
Multiparous (excl prev CS), single vertex, ≥37 wk spontaneous labour -3	Multip Term Spontaneous
Multiparous (excl prev CS), single vertex, ≥37 wk, Induced or CS wi No labour -4	Multip Term Elective
Previous CS, single vertex, ≥37 wk -5	Previous CS
All nulliparous breeches -6	Nullip Breech
All multiparous breeches -7	Multip Breech
All multiple pregnancies -8	All multiples
All 'other' lies -9	Transverse lies
All single vertex, ≤ 36 wk -10	Preterm

Caesarean rates were calculated for all births for each 10-group classification category per year. Due to the increase in maternal age over the years in order to compare CS rates, direct age-standardised rates (ASR) of CS were calculated. The population of women of reproductive age (10-54 years) in New South Wales in 2007 was used as the standard and was obtained from the Australian Bureau of Statistics [32]. Trend analyses were performed using the Joinpoint Regression Program provided by the US National Cancer Institute http://srab.cancer.gov/joinpoint. All other statistical analysis was performed using SAS software, version 9.1 (SAS Institute Inc, Cary, NC). Average Annual Percentage Change (APC) for caesarean rates were calculated and presented with 95% confidence limits. To determine whether changes in maternal and pregnancy characteristics explained the trend in CS rates we used logistic regression and compared crude (univariate) and adjusted odds ratios (ORs) of CS for each year compared to 1998, with statistical significance reported at the *P *< 0.05 (two-tailed) level. In addition the relationship between 10-group classification categories and overall CS rate for each hospital group was examined using linear regression analyses [27].

## Results

The total number of women who gave birth from 1998-2008 was 965,702, including 151,516 who had a primary caesarean section (61.9%) and 93,306 who had a repeat caesarean section (38.1%). The overall CS rate from 1998-2008 was 25.4 per 100 births and increased from 19.1 per 100 births in 1998 to 29.5 per 100 births in 2008 (Table 2). The rate of primary caesareans in NSW increased from 11.9 per 100 births in 1998 to 17.8 per 100 births in 2008 (P-trend < 0.001, average annual increase 4.3% (95%CI 3.0-5.7%), with the largest increase occurring among term births. The rate of repeat caesareans increased from 7.8 per 100 births to 12.0 per 100 births from 1998 to 2008 (P trend < 0.001, average annual increase 4.8% (95% CI 3.9-5.7%). Adjustment for maternal and pregnancy factors had little impact on the odds ratio for CS in each year, so the increase in CS delivery over time was maintained (P-trend < 0.001, Table 2)

**Table 2:** CS delivery by year*, NSW, 1998- 2008

	No caesarean (row %) (n = 720,880)	Caesarean (row %) (n = 244,822)	Crude OR	Adjusted OR* (95% CI)
Year of delivery				
1998	68,856 (80.9)	16,216 (19.1)	Referent	Referent
1999	69,055 (80.3)	16,912 (19.7)	1.04 (1.02-1.07)	1.04 (1.01-1.07)
2000	68,004 (78.7)	18,456 (21.3)	1.15 (1.13-1.18)	1.18 (1.14-1.22)
2001	64,499 (76.4)	19,880 (23.6)	1.31 (1.29-1.34)	1.36 (1.32-1.40)
2002	63,532 (75.1)	21,055 (24.9)	1.40 (1.37-1.44)	1.46 (1.41-1.50)
2003	62,468 (73.5)	22,564 (26.5)	1.53 (1.50-1.57)	1.58 (1.53-1.62)
2004	61,384 (72.8)	22,904 (27.2	1.58 (1.55-1.62)	1.62 (1.58-1.67)
2005	64,063 (71.9)	25,077 (28.1)	1.66 (1.62-1.70)	1.69 (1.64-1.74)
2006	64,981 (71.2)	26,334 (28.8)	1.72 (1.68-1.76)	1.79 (1.74-1.84)
2007	67,152 (71.0)	27,450 (29.0)	1.74 (1.70-1.78)	1.74 (1.69-1.79)
2008	66,886 (70.5)	27,974 (29.5)	1.78 (1.74-1.82)	1.76 (1.71-1.81)

*adjusted for year of delivery, hospital group, maternal age, parity, maternal hypertension, maternal diabetes, maternal smoking during pregnancy, previous CS, plurality, onset of labour, birth presentation, pre-term birth, size at birth(< 10^th ^and > 90^th ^percentile)

Table 3 shows the rates of CS births by Robson's 10-group classification between 1998-2008. Previous CS group (ASR 8.4 per 100 births, 32.5% of all CS) made the greatest contribution to the total CS rate. Nullip Term Elective deliveries had the second highest contribution to the CS rate (ASR 4.9 per 100 births, 20.6% of all CS) and then Nullip Term Spontaneous (ASR 2.9 per 100 births, 12.3% of all CS). Consequently nulliparae at term made up one-third of the overall CS and previous CS group made up another one-third.

**Table 3:** Age-standardised Rates of CS for 10-group classification and Hospital Groupings, NSW, 1998-2008

Groupings	Caesarean Rate in each group (%)	Contribution made by each group to overall CS rate % (denom = 965,702)	ASR (per 100 deliveries) of contribution made to overall CS rate	APC (95% Upper and Lower CL) in ASR (%)
10-Group Classification				
Nulliparous, single vertex, ≥37 wk, spontaneous labour -1	29,961/225,216 (13.3)	3.1	2.9	3.5 (1.8; 5.2)*
Nulliparous, single vertex, ≥37 wk, Induced or CS wi No Labour-2	50,421/127,004 (39.7)	5.2	4.9	6.8 (5.5; 8.2)*
Multiparous (excl prev CS), single vertex, ≥37 wk spontaneous labour -3	6,146/280,506 (2.2)	0.6	0.6	1.1 (-1.3; 3.6)
Multiparous (excl prev CS), single vertex, ≥37 wk, Induced or CS wi No labour -4	17,736/121,911 (14.6)	1.8	1.8	4.0 (1.9; 6.2)*
Previous CS, single vertex, ≥37 wk -5	79,480/104,160 (76.3)	8.2	8.4	5.3 (4.8; 5.8)*
All nulliparous breeches -6	17,410/19,294 (90.2)	1.8	1.7	0.8 (-0.3; 2.0)
All multiparous breeches -7	14,775/17,730 (83.3)	1.5	1.5	-1.0 (-2.8; 0.7)
All multiple pregnancies -8	8,651/15,163 (57.0)	0.9	1.8	3.6 (1.3; 5.9)*
All 'other' lies -9	5,430/6,979 (77.8)	0.6	0.6	-2.8 (-4.6; -1.1)*
All single vertex, ≤ 36 wk -10	14,159/46,734 (30.3)	1.5	1.4	4.0 (3.6; 4.4)*

*Indicates significant at p < 0.05 level

Abbreviations: ASR = age-standardised rate; APC = annual percentage change

**Figure 1:**
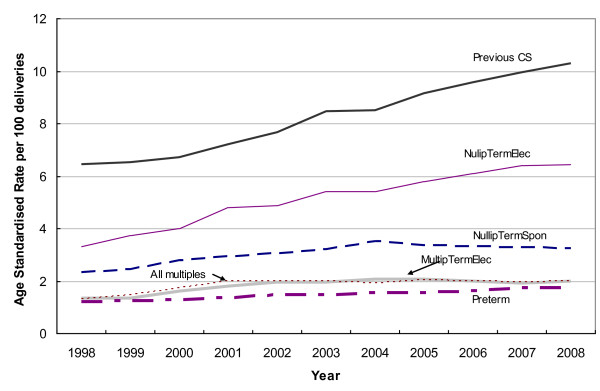
Age-standardised rate of CS delivery per year, 10-group classification, NSW, 1998-2008*. * Only groups for which there was a significant increase in annual percentage change are presented.

With the exception of Multip Term Spontaneous, Multip breech and Transverse lies, all other CS groups had significantly increased annual percentage changes (Figure 1). Nullip Term Elective (APC 6.8, 95% CI 5.5-8.2) and previous CS (APC 5.3%, 95% CI 4.8-5.8) had the largest annual increases, but were not significantly different to the changes per year of the Nullip Term Spontaneous (APC 3.5%, 95% CI 1.8-5.2%) and Multip Term Elective (APC 4.0, 95% CI 1.9-6.2%) groups. The combined Nullip Term group had an average annual increase of 5.6% (95% CI 4.2-6.9%).

Among the previous CS group, the main contributor to the increased CS rate was CS prior to labour, with rates of CS following spontaneous and induced labour remaining stable over the study period. In the Nullip Term Elective group Caesarean section after induction of labour also increased, but less dramatically.

CS rates varied by hospital type: tertiary ASR = 9.6/100 births, APC = 4.1% (2.9%-5.2%); private ASR = 7.9, APC = 5.6% (2.5%-8.9%); district ASR = 6.1, APC = 1.4% (0.5%-3.4%); and small rural ASR = 2.2, APC = 3.5% (2.4%-4.7%). The relationship between the NullipTerm CS rate and the overall CS rate in each hospital group was highly correlated (Adj R^2 ^= 0.99, p < 0.001), indicating that 99% of the variation in the overall CS rate for each hospital group may be explained by the combined NullipTerm CS rate.

## Discussion

From 1998 to 2008 the CS rate in NSW increased from 19.1 to 29.5 per 100 births, giving an overall rate of 25.4 per 100 births. This CS rate is similar to rates reported elsewhere in Australia which range from 28.0 in Tasmania to 33.1 in Queensland [1]. When compared to caesarean rates around the world, this CS rate is higher than Norway (13.9)[29], similar to Asian countries (27.3)[33] but lower than that reported in the USA (31.1)[4] The increase in the age-standardised rate in NSW was in both primary and repeat CS, similar to the trends seen in the USA. However, despite small increases in preterm CS, the majority of the increase in the primary CS rate in NSW occurred in term births. This is in contrast to the USA where increases appear to be similar across all gestational age categories [4]. The increase in the repeat CS delivery rate in New South Wales was predominantly driven by CS prior to the onset of labour.

The lack of explanation of the increased CS rate by known maternal or pregnancy characteristics in this study is similar to findings from a population-based study conducted in Western Australia [34]. O'Leary et al showed an increase in elective and emergency CS rates which could not be totally explained by demographic or obstetric characteristics, leading the authors to postulate the possibility of maternal request playing an increasing role in the increase in CS in Western Australia [34]. A recent survey of Australian obstetricians concluded that in 2006, 17.3% of prelabour caesarean sections may have been undertaken on maternal request, however corresponding trend data over the study period are not available [35]. Another potential contributing factor to increases in CS rates may be the high and increasing rates of obesity (24% of Australian women compared to an average of 16% across 139 countries) [36]. While population-health datasets, such as those used in O'Leary's study and our own study, allow investigation of wider population demographic and obstetric factors that may be associated with CS rates, they often do not have detailed data on patient factors such as maternal request, obesity or pregnancy weight gain, or service delivery issues such as midwifery models of care or obstetric training and practice.

### Robson groupings

When examining CS rates classified according to the 10-group classification, the main contributors to the overall CS rate in NSW were the Previous CS and Nullip Term Elective groups, which is similar to other studies [2,17,27]. The largest annual increases also occurred for the Nullip Term Elective and Previous CS groups. This was the first time, to the authors' knowledge, that annual trends for age-standardised rates have been investigated for the 10-group classification in an attempt to ascertain which clinically relevant groups were contributing to the increasing CS rate over time. The combined Nullip Term group had an average annual increase of 5.6%, reflecting the increase as described by the primary CS rate.

Using methods based on a multi-institution international study[27], 99% of the variance in CS rate in each hospital group could be attributed to the rate of CS occurring in Nullip Term women. This was in agreement with the previous study and it may be concluded that if the CS rate is reduced in nulliparous women with term singleton vertex presentations, then the future CS rate may be reduced. The contribution of the Nullip Term group to the overall CS rate was 33%, similar to rates reported in domestic and overseas studies [2,17,27].

The CS rate in the Previous CS group was over 76% and its contribution to all births was 8.2%, similar to that reported elsewhere [2,17]. This rate, combined with the fact that the increase over time was predominately due to CS prior to labour, may indicate that vaginal birth after CS is not being considered by obstetricians and/or women. Together the contributions of the Nullip Term and Previous CS groupings to the overall CS rate was over 65%, slightly higher than in hospital-based studies [17,27].

One of the limitations of the Robson classification is that women undergoing elective induction of labour are classified in the same group as women undergoing caesarean section before labour (Nullip Term Elective and MultipTerm Elective, groups 2 and 4). Caesarean sections for maternal request are thus included in the same groups as induction of labour for recognised obstetric indications such as post dates, elective caesarean section for placenta praevia, and elective inductions for maternal or obstetric care-giver convenience. Given the reported increase in inductions of labour that may well be for non-obstetric reasons[37], as well as the increase in CS rates that are not explained by known and collected obstetric factors, it is important that future research can identify and monitor trends in and reasons for induction of labour and caesarean section, and associated outcomes.

## Conclusions

Nearly two-thirds of the overall CS rate is due to primary caesarean sections and the rate of both primary and repeat CS is increasing with time. Given that 76% of those with a previous CS who deliver at term have a CS and that 99% of the overall variation in the CS rate may be explained by nulliparous, cephalic term CS, efforts to reduce the overall CS rate should be focussed on reducing the primary CS rate.

Australia has low maternal and perinatal mortality rates by international standards [3]. The reasons for rising caesarean section rates are complicated and multifactorial. However, it is important to consider that a caesarean section has major implications for a woman's health and wellbeing in the current pregnancy, potential morbidity such as increased risk of placenta praevia and accreta in subsequent pregnancies and has significant cost implications for the health care system. In the current context of maternity service provision reform in Australia, it is important to ensure continuing low rates of maternal and perinatal morbidity and mortality.

## Competing interests

The authors declare that they have no competing interest.

## Authors' contributions

ES, JF and CR designed the study, ES conducted the analyses and wrote the manuscript, JF, AS, CR and JM contributed to interpretation of analyses and writing the manuscript. All authors approved the final manuscript.

## Pre-publication history

The pre-publication history for this paper can be accessed here:

http://www.biomedcentral.com/1471-2393/11/8/prepub
